# Outcome of Radial Nerve Palsy in Closed Humeral Shaft Fractures Treated Operatively and Expectantly

**DOI:** 10.1016/j.jhsg.2026.101091

**Published:** 2026-08-07

**Authors:** Kareme D. Alder, Nicholas Pulos, Alexander Y. Shin

**Affiliations:** ∗ Department of Orthopedic Surgery, Mayo Clinic, Rochester, MN

**Keywords:** Humeral shaft fracture, Nerve recovery, Nonsurgical treatment, Open reduction internal fixation, Radial nerve palsy

## Abstract

**Purpose:**

Recent trends favor earlier surgical intervention for humeral shaft fractures, based on the idea that operatively treated humerus fractures with associated radial nerve palsy may result in an earlier return of nerve function. The primary aim of this study was to evaluate whether the timing of radial nerve recovery differs between patients managed expectantly and operatively.

**Methods:**

A retrospective review of closed humeral shaft fractures over a 12-year period was conducted. Patients with an associated radial nerve palsy were identified. Analysis focused on the time to onset of radial nerve motor recovery.

**Results:**

A total of 15 patients with humeral fractures with radial nerve palsy were included in the final cohort: eight were treated with expectant management and seven underwent acute open reduction internal fixation. All patients exhibited complete radial nerve recovery within 53 weeks. The mean onset of initial radial nerve recovery was 8.4 weeks, with full recovery achieved at an average of 32.6 weeks. Statistical analysis demonstrated no significant difference in the onset of (6.4 vs 10.8 weeks; *P* = .28) and full (35.7 vs 29.9 weeks; *P* = .6) recovery between patients treated conservatively and operatively.

**Conclusions:**

Radial nerve palsy associated with closed humeral shaft fractures have a high rate of spontaneous nerve recovery, with similar onset and full recovery of nerve function despite the treatment option chosen. Closed humeral shaft fractures with concomitant radial nerve palsy should not be an indication for operative treatment with the aim of decreasing the time to nerve recovery.

**Type of study/level of evidence:**

Therapeutic IV.

Radial nerve palsy following a diaphyseal fracture of the humerus is the most common peripheral nerve injury associated with a long bone fracture, with an overall prevalence of 12.3%.^1^^,^^2^ Injuries of the radial nerve frequently occur because of its intimate location within the spiral groove of the posterior humerus that predisposes the nerve to contusion, crush, entrapment, or laceration following fracture.^3^ The radial nerve may also be injured during closed manipulation and immobilization or open reduction internal fixation.^4^, ^5^, ^6^ Radial nerve palsies have a high rate of spontaneous recovery without operative intervention, reported at 77.2%.^2^

The treatment of open humeral shaft fractures with a concomitant radial nerve palsy are unanimously treated with urgent debridement, irrigation, nerve exploration/repair, and open reduction internal fixation.^7^, ^8^, ^9^ Algorithms for treating closed humeral shaft fractures with associated radial nerve palsy vary; traditional dogma advocates that patients with closed humeral shaft fractures and radial nerve palsy should initially be treated conservatively with 3 to 6 months of expectant management.^9^, ^10^, ^11^, ^12^, ^13^ Surgeons supporting initial expectant management propose that closed humeral shaft fractures with radial nerve palsy should not be explored, given the high rate of spontaneous nerve recovery and the risk of secondary radial nerve injury.^10^^,^^12^, ^13^, ^14^, ^15^ Conversely, there is a trend of early operative intervention where proponents cite increased technical difficulty in delayed cases secondary to fracture callus and scar development as a rationale for early operation.^16^

Current literature has begun to advocate for a paradigm shift toward earlier nerve exploration in all humeral shaft fractures,^2^^,^^17^, ^18^, ^19^ with reports of greater and possibly earlier rates of radial nerve recovery following acute open reduction internal fixation and nerve exploration.^2^^,^^17^^,^^18^ However, most of these studies have heterogeneous patient populations, including open and closed injuries.^2^^,^^19^ Additionally, few, if any, studies compare the natural history of radial nerve recovery in conservatively and operatively treated patients with closed humeral shaft fractures to determine if there is a benefit to time to recovery.^12^^,^^18^, ^19^, ^20^, ^21^, ^22^, ^23^ The primary objective of this investigation was to delineate the timeline of radial nerve regeneration in patients with closed humeral shaft fractures accompanied by radial nerve palsy, managed through both conservative and surgical interventions. The secondary objective was to evaluate the influence of injury mechanism and fracture classification on the trajectory of radial nerve recovery.

## Materials and Methods

Mayo Clinic Institutional Review Board waived the requirement of informed consent, as no defining patient data were used or collected, and informed consent would have been impractical to obtain. All procedures followed were in accordance with the ethical standards of the responsible committee on human experimentation (institutional and national) and with the Helsinki Declaration of 1975.

After institutional review board approval (IRB#25-001022), a retrospective review was performed of all adult patients who sustained a closed diaphyseal humeral shaft fracture with concomitant radial nerve palsy between 2010 and 2022, treated at a level 1 trauma and tertiary care academic institution. Patients who did not have at least one follow-up at our institution, had secondary radial nerve palsy, had multiple nerve injuries, and/or associated injuries of the ipsilateral arm were excluded. Patient demographics, injury mechanism, fracture location/pattern (ie, spiral, transverse, or oblique), fracture treatment modality, surgical details, motor unit strength (ie, thumb extension, finger extension, and wrist extension), sensation, and time to early and full nerve recovery were recorded.

All patients treated surgically underwent humeral fixation by a fellowship-trained orthopedic traumatologist. Details regarding motor unit strength, sensation, and signs of early and full recovery were documented based on clinical examination performed by a fellowship-trained hand surgeon/peripheral nerve surgeon. Motor strength was recorded using the modified British Medical Research Council (mBMRC) Scale for Muscle Strength (Table 1).^24^ Sensation was subjectively graded as absent, diminished, or normal per patient report. The time to first sign of radial nerve recovery was determined as the date from injury at which a patient had a clinical examination with a recorded mBMRC muscle strength greater than zero. In contrast, time to full nerve recovery was determined as the date of either full muscle strength return (mBMRC grade >4) of the wrist, finger, and thumb extensors or discharge from the clinic with a fully functional wrist, finger, and thumb extension as reported by the injured patient.

**Table 1:** Modified British Medical Research Council Muscle Strength Scale

Grade	Degree of Muscle Strength	Clinical Terms
0	No palpable contraction	Nil
1	Palpable contraction	Trace
2	Movement with gravity eliminated	Gravity eliminated
3	Movement through the full arc of motion against gravity	Antigravity
4	Full range of motion against gravity plus resistance	Near normal
5	Normal strength	Normal

### Statistical analysis

Descriptive statistics were used to summarize patient demographic information, injury characteristics, and treatment modality. Patients were subsequently divided into expectant management (ie, nonsurgical management and delayed open reduction internal fixation) and operative management. Time to first recovery and full recovery were compared using Welch’s *t* test. Motor recovery based on treatment modality and mechanism of injury was compared using Welch *t* test. Welch *t* test data are reported as the mean with a 95% confidence interval for the mean difference. Differences in radial nerve recovery based on fracture pattern were compared using analysis of variance. Significance was set at *P* < .05 for all statistical tests.

## Results

### Patient demographics

**Figure 1:**
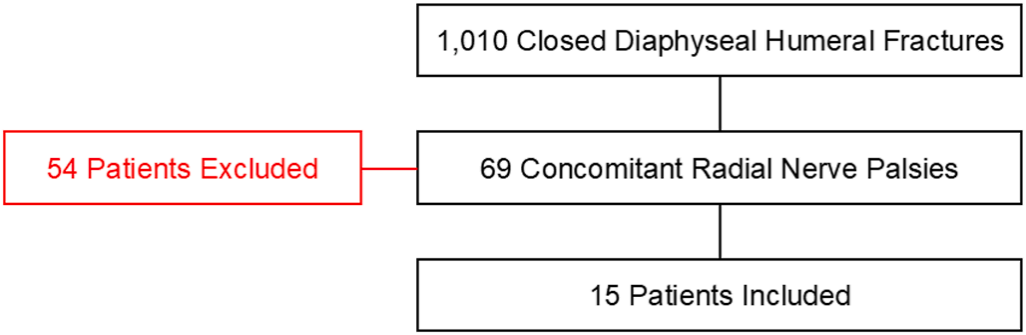
Flow chart for patient inclusion and exclusion. Between 2010 and 2022, a total of 1,010 patients were identified with a closed humeral shaft fracture. Within this cohort of patients, 69 patients were found to have a concomitant radial nerve palsy. Fifty-four patients were excluded for no follow-up at our institution (n = 30), secondary radial nerve palsy (n = 21), and multiple nerve injuries (n = 3). A total of 15 patients were left for final analysis.

A total of 1,010 patients with an acute diaphyseal humeral shaft fracture were identified, with 69 (6.8%) having concomitant radial nerve palsy at the time of presentation. After applying exclusion criteria, 15 patients were included for final analysis (Fig. 1). Two patients were lost to follow-up after demonstrating the onset of radial nerve recovery. The mean age was 51 ± 24.4 years, with 4 (26.7%) men and 11 (73.3%) women. The average body mass index was 30.9 kg/m^2^. Most patients were right-hand dominant (n = 14; 93.3%). One patient had type 2 diabetes mellitus that was well-controlled (HbA1c below 7.0%), and one patient had a diagnosis of polymyalgia rheumatica. No patient was on immunosuppressive or chemotherapeutic medications. No patient had a pre-existing diagnosis of neuropathy or nerve injury. Most patients included in the study held an office/administrative job (n = 7; 46.7%). Of the remaining patients, 3 (20%) were retired, 2 (13.3%) worked as manual laborers/farmers, 2 (13.3%) were unemployed, and 1 (6.7%) was a student. Within the cohort, three patients actively used nicotine-containing products and two carried a diagnosis of alcohol use disorder.

### Injury characteristics

All patients included in the study sustained a closed midshaft diaphyseal humeral fracture without significant comminution, with most injuries involving the dominant upper extremity (n = 10; 66.7%). Fractures were the result of a ground level fall (n = 8; 53.3%), high-energy motor vehicle trauma (n = 5; 33.3%), and low-energy sporting events (n = 2; 13.3%). No patient with a low-energy mechanism sustained concomitant injuries, while four patients involved in motor vehicle trauma were polytraumatized. Of the fractures sustained, 8 (53.3%) were spiral, 4 (26.7%) transverse, and 3 (20%) oblique.

### Treatment modality

Six (40%) patients underwent nonsurgical treatment based on a collaborative decision between the patient and surgeon—five patients underwent Sarmiento bracing and one patient was treated in a hanging arm cast. Seven patients underwent acute open reduction internal fixation with a locking plate, given patient and surgeon preference for immediate upper extremity weightbearing (n = 6) and/or early loss of reduction (n = 1) at an average of 8.7 ± 3.7 days. Two patients underwent delayed open reduction internal fixation with a locking plate at 66 and 87 days for nonunion after early nerve recovery was noted. Patients underwent either an anterolateral (n = 3; 33.3%) or Gerwin-Hotchkiss^25^ (n = 6; 66.7%) approach to the humerus, with the radial nerve visualized, mobilized/neurolyzed, and protected before locking plate osteosynthesis. All radial nerves were found to be in continuity. All patients went on to uneventful humeral shaft fracture union after treatment. No patient required tendon transfer for persistent radial nerve palsy. Patient demographics, injury characteristics, and treatment modalities are recorded in Table 2.

**Table 2:** Patient Demographics, Injury Characteristics, and Treatment Modality

Sex, n (%)	
M	4 (26.7)
F	11 (73.3)
Age, mean ± SD (y)	51 ± 24.4
BMI, mean ± SD (kg/m^2^)	30.9 ± 9.0
Race/ethnicity, n (%)	
White	12 (80)
Asian or Pacific Islander	0 (0)
Black	2 (13.3)
Hispanic or Latino	1 (6.7)
Hand dominance, n (%)	
R	14 (93.3)
L	1 (6.7)
Side of injury, n (%)	
R	9 (60)
L	6 (40)
Dominant hand injury, n (%)	
Dominant hand	10 (66.7)
Nondominant hand	5 (33.3)
Mechanism of injury, n (%)	
Ground level fall	8 (53.3)
High-energy trauma	5 (33.3)
Sporting injury	2 (13.3)
Fracture pattern, n (%)	
Spiral	8 (53.3)
Transverse	4 (26.7)
Oblique	3 (20)
Treatment, n (%)	
Nonsurgical	6 (40)
Acute open reduction internal fixation	7 (46.7)
Delayed open reduction internal fixation	2 (13.3)

### Radial nerve recovery

**Figure 2:**
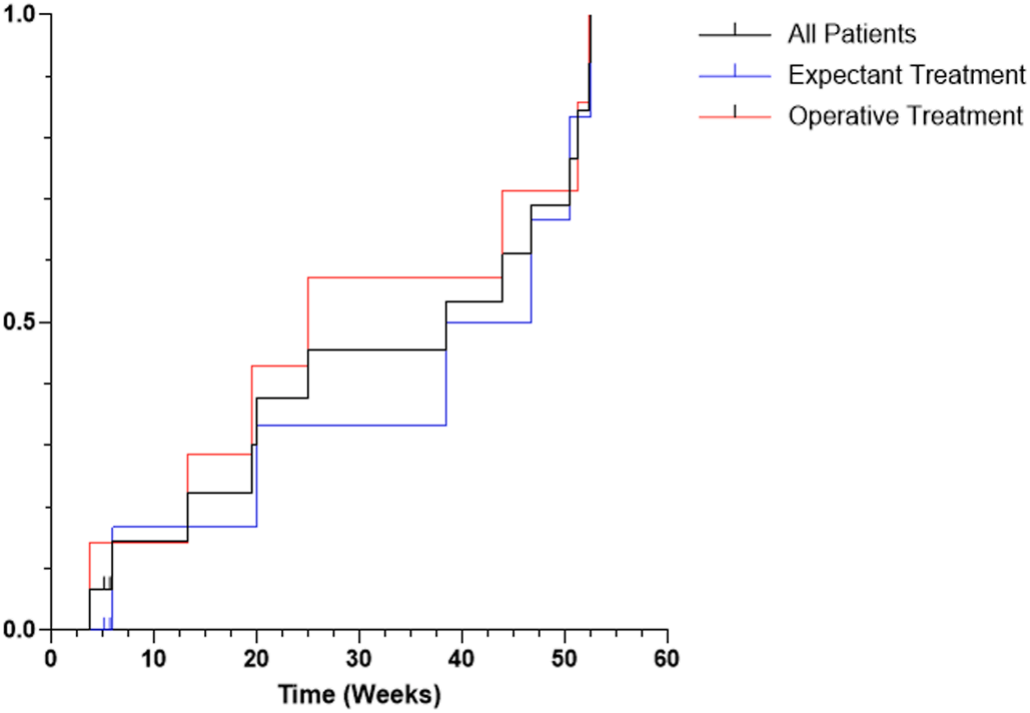
Full radial nerve recovery. All radial nerve palsies went on to full recovery. All patients had normal motor and sensory recovery. All patients had full recovery within 53 weeks.

**Figure 3:**
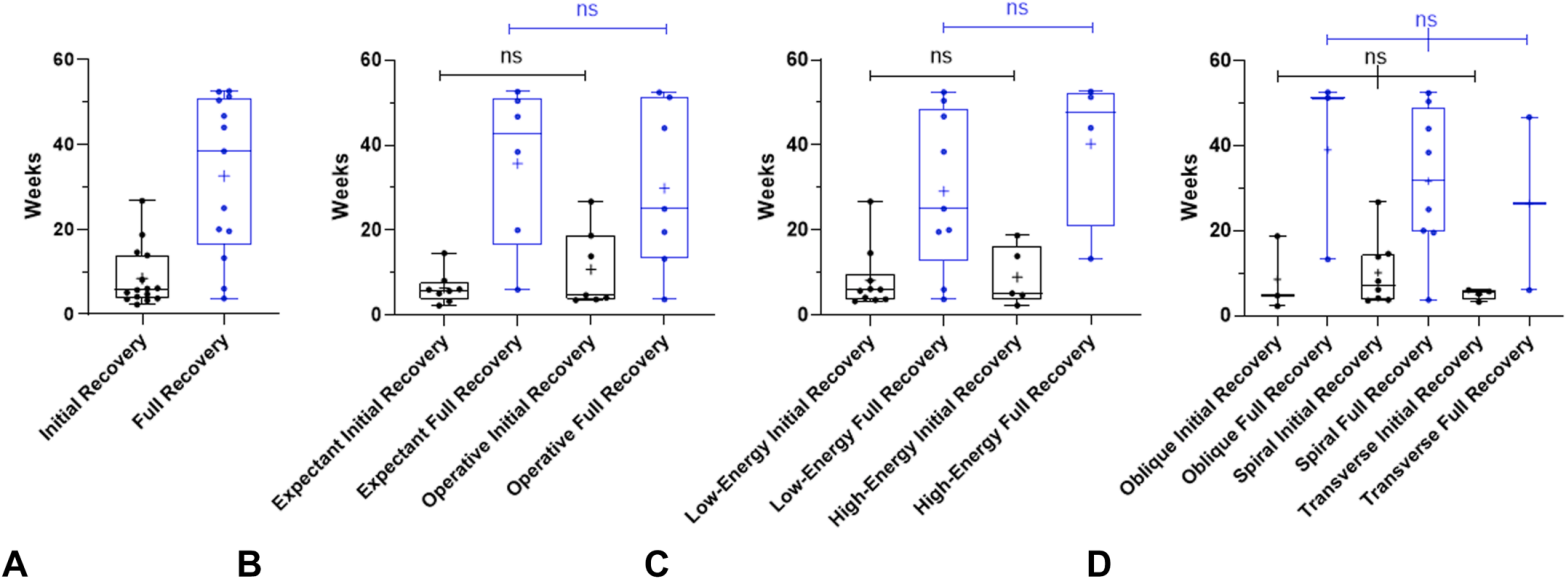
Comparison of treatment, mechanism energy, fracture pattern, and radial nerve recovery. **A** The average time to first sign of radial nerve recovery was 8.4 ± 7.0 weeks, with full recovery occurring at an average of 32.6 ± 18.5 weeks for all patients. **B** There were no statistically significant differences between the time to first sign of neurologic recovery (6.4 vs 10.8 weeks, 95% CI [−13.0, 4.3]; *P* = .28) or full neurologic recovery (35.7 vs 29.9 weeks, 95% CI [−17.6, 29.2]; *P* = .6] in patients treated with expectant management or acutely operatively, respectively. **C** There were no statistically significant differences in time to first sign of radial nerve recovery (8.2 vs 8.9 weeks, 95% CI [−8.2, 9.6]; *P* = .85) and full recovery (29.1 vs 40.3 weeks, 95% CI [−16.1, 38.4]; *P* = .35) based on injury mechanism stemming from low- or high-energy trauma, respectively. **D** There were no statistically significant differences between early radial nerve recovery secondary to oblique (8.6 ± 8.9 weeks), spiral (10.1 ± 8.0 weeks), or transverse (5.0 ± 1.2 weeks) fracture pattern (*F*-value = 0.68). No statistically significant differences existed between full radial nerve recovery based on oblique (39.0 ± 22.3 weeks), spiral (31.7 ± 17.3 weeks), or transverse (26.3 ± 28.9 weeks) fracture pattern (*F*-value = 0.27). The symbol + represents the mean value for each data set. Significance set at *P* < .05 for all statistical tests.

All patients initially presented with absent radial nerve motor or sensory function (mBMRC grade 0) with respect to wrist extension (extensor carpi radialis longus, extensor carpi radialis brevis), finger extension (extensor indicis proprius, extensor digitorum communis, extensor digiti minimi), and thumb extension (extensor pollicis longus) and diminished or absent sensation in the sensory branch of the radial nerve at presentation. All patients ultimately fully recovered their radial nerve (Fig. 2). The average time to first sign of radial nerve recovery was 8.4 ± 7.0 weeks (Fig. 3A). Wrist extension was the muscle group with the earliest motor recovery documented, with an average mBMRC muscle strength of 2 ± 1.6 at the first date of reported recovery. Complete recovery occurred at an average of 32.6 ± 18.5 weeks for all patients (Fig. 3A). All patients reported normal sensory branch of the radial nerve sensation at the time of documented full recovery.

There were no statistically significant differences between the time to first sign of neurologic recovery (6.4 vs 10.8 weeks, 95% CI [−13.0, 4.3]; *P* = .28) or full neurologic recovery (35.7 vs 29.9 weeks, 95% CI [−17.6, 29.2]; *P* = .6) in patients treated with expectant management or acutely operatively, respectively (Fig. 3B). Wrist extension was the first sign of recovery in both groups, with no significant statistical difference between the Medical Research Council muscle strength of expectantly and operatively managed patients (1.4 vs 2.7, 95% CI [−3.1, 0.41]; *P* = .12). There were no statistically significant differences in time to first sign of radial nerve recovery (8.2 vs 8.9 weeks, 95% CI [−8.2, 9.6]; *P* = .85) and full recovery (29.1 vs 40.3 weeks, 95% CI [−16.1, 38.4]; *P* = .35) based on injury mechanism stemming from low- or high-energy trauma, respectively (Fig. 3C). Similarly, there were no statistically significant differences between early radial nerve recovery secondary to oblique (8.6 ± 8.9 weeks), spiral (10.1 ± 8.0 weeks), or transverse (5.0 ± 1.2 weeks) fracture pattern (*F*-value = 0.68). No statistically significant differences existed between full radial nerve recovery based on oblique (39.0 ± 22.3 weeks), spiral (31.7 ± 17.3 weeks), or transverse (26.3 ± 28.9 weeks) fracture pattern (*F*-value = 0.27) (Fig. 3D).

## Discussion

Radial nerve palsy is a common sequelae following humeral shaft fracture.^1^ In patients with open diaphyseal humeral fractures, definitive treatment is accomplished with urgent operative debridement, irrigation, nerve exploration, and open reduction internal fixation of the fracture, given the high incidence of nerve laceration or interposition within the fracture bed.^7^ Conversely, closed humeral shaft fractures with radial nerve palsy are traditionally treated with expectant management.^9^, ^10^, ^11^, ^12^, ^13^ There is a growing movement for early radial nerve exploration in all humeral shaft fractures.^2^^,^^17^, ^18^, ^19^ However, there is a paucity of data regarding the early and full recovery timing of radial nerve recovery in expectantly managed and operatively treated closed fractures.

In the present study, we assessed the natural history of radial nerve palsy in closed diaphyseal humeral shaft fractures treated with expectant management and acute operative fixation of the humerus and radial nerve exploration. All patients had full recovery of their radial nerve palsy. In all patients with a radial nerve palsy sustained after a closed humeral shaft fracture, the average time of first sign of recovery was 8 weeks, while full recovery was 33 weeks. Entezari et al^22^ illustrated early recovery in radial nerve palsy to occur at a median of 9 weeks and full recovery at 33 weeks in a cohort of conservatively and operatively managed patients. Lang et al^21^ found that the onset of radial nerve recovery in primary radial nerve palsy following humeral shaft fracture was at an average of 10.5 weeks and full recovery at 27 weeks. A systematic review by Shao et al^20^ found early radial nerve recovery to occur at 7 weeks. However, the work of Entezari et al, Lang et al, and Shao et al had heterogeneous patient cohorts inclusive of patients with open fractures, partial radial nerve palsy, and/or multiple peripheral nerve injuries. We establish an expected timing of first onset and full recovery of patients with radial nerve palsy sustained in closed humeral shaft fractures.

Our study found no statistically significant differences between patients with radial nerve palsy in the context of a closed humeral shaft fracture treated expectantly and operatively, with average early recovery at 6 weeks and 11 weeks and full recovery at 36 and 30 weeks, respectively. Entezari et al found that the median time to first sign of recovery in expectantly managed patients was 7 weeks, while operatively managed patients showed recovery at 9 weeks—though they did not report on the timing of full recovery in these groups. Gomez et al^23^ illustrated that primary radial nerve palsies sustained during closed humeral shaft fractures treated operatively had a median time to early nerve recovery of 16 weeks and full recovery of 36 weeks, but did not compare nonsurgically treated patients. Keighley et al^18^ demonstrated that 10 patients with radial nerve palsy and a closed humeral shaft fracture had a 100% radial nerve recovery rate at a mean of 23 weeks after acute neurolysis and open reduction internal fixation. Korompilias et al^13^ identified that 13 nonsurgically treated patients with radial nerve palsy and closed humeral shaft fracture had full return of nerve function at an average of 12 weeks, while 10 patients who underwent neurolysis with an intact nerve recovered at an average of 22 weeks—they did not assess the timing of the first onset of radial nerve recovery. We establish that there is no significant difference in the time to first onset and full nerve recovery in expectantly and operatively treated radial nerve palsy sustained in closed humeral shaft fractures.

The present study did not find statistically significant differences between the time to first sign of radial nerve recovery and full recovery based on the energy of the injury mechanism. We demonstrated the onset of radial nerve recovery at an average of 8 weeks and 9 weeks and full recovery at 29 and 40 weeks in low- and high-energy trauma, respectively. Ring et al^8^ showed that a heterogeneous group of patients with closed and open radial nerve palsy sustained via a high-energy mechanism had an onset of recovery at an average of 7 weeks and full recovery at 24 weeks, which is similar to the overall recovery rates of all radial nerve injuries. Conversely, the work of Venouziou et al^26^ suggests that radial nerve palsies sustained as part of a high-energy humeral shaft fracture require an increased period of time to fully recover. While multiple studies have identified that Holstein-Lewis, transverse, and spiral fractures are associated with radial nerve palsy, there is limited information regarding recovery timing based on fracture pattern.^1^^,^^20^ In the present study, we did not find any significant differences in nerve recovery based on the closed humeral shaft fracture pattern.

The indication for surgical treatment of a closed humerus fracture associated with an intact radial nerve palsy should not be the nerve palsy, since they generally recover without surgery. In a recent systematic review, a heterogeneous population inclusive of closed/open injuries and primary/secondary palsy demonstrated a 77.2% spontaneous recovery rate.^2^ The recovery has been shown to be higher in populations with purely closed injuries,^13^^,^^27^ with a 100% recovery rate in our series of closed injuries. While the rate of nerve laceration or interposition has been reported to be 64% in open fractures, most closed injuries are secondary to neuropraxia, with laceration found in 12% of patients.^7^^,^^10^ Concern for the rare lacerated nerve may be evaluated with noninvasive modalities. Ultrasonography has been shown to have a 92% concordance rate between imaging and intraoperative findings regarding radial nerve continuity—with a lacerated nerve acutely necessitating operative exploration and/or grafting.^28^ We suggest consideration of ultrasonography in closed humeral shaft fractures with an associated radial nerve palsy to assess nerve continuity before expectant management. If a nerve is in continuity, our data suggest that there is no decreased time to onset or full recovery of an expectantly or operatively treated radial nerve palsy in closed humeral shaft fractures. Acute operative intervention is not without risk and may result in anesthesia complications, infection, or secondary nerve palsy. Shoji et al^29^ demonstrated that there is no increased risk of iatrogenic radial nerve injury with delayed operative intervention.

Furthermore, current guidelines suggest that patients presenting with radial nerve palsy should be evaluated for potential surgical intervention, including nerve exploration, grafting, or nerve transfers within a 3- to 6-month timeframe.^9^ However, in our series, less than 50% of patients fully recovered at 24 weeks, with 7% yet to demonstrate early nerve recovery. Using 6 months as the timing for nerve surgery may predispose unwarranted surgery based on the natural recovery history. Expectant delay in operative intervention for a radial nerve palsy may spare a patient an unnecessary surgery for a nerve that would recover at the same rate, within the same period, and without surgical intervention. Last, in the rare setting in which radial nerve regeneration fails to occur, tendon transfers for radial nerve palsy have a high success rate and are efficacious in remote palsies.^30^

We recognize the limitations of this study. It is retrospective, with the biases typical of this design. Patients were excluded because they did not have follow-up at our tertiary institution after their initial injury. This loss of patients resulted in a smaller sample size, which may have increased the likelihood of a type II error. The retrospective approach led to varying follow-up times. Standardized follow-up periods could have enabled earlier detection of the onset and resolution of radial nerve palsy. Retrospective analysis can lead to incomplete or inaccurate medical documentation. We confirmed that each patient had a complete radial nerve palsy at presentation to our emergency department. To ensure accuracy, we only assessed documentation made by a fellowship-trained orthopedic traumatologist or hand/peripheral nerve surgeon during data collection, cross-referencing data whenever possible.

## Conclusions

In conclusion, we identify an average time to the onset and full recovery of radial nerve palsies in closed humeral shaft fractures and demonstrate no difference in timing between expectant and operative interventions. Additionally, we demonstrate no differences in radial nerve recovery timing of recovery in low- and high-energy closed injuries or based on fracture patterns. Our data suggest that patients with radial nerve palsy following a closed humeral shaft fracture should be treated expectantly, given the high rate of recovery, and that operative intervention should not be indicated with the goal of decreasing the time of nerve recovery.

## Conflicts of Interest

No benefits in any form have been received or will be received related directly to this article.
